# Release of Pro-Inflammatory/Angiogenic Factors by Retinal Microvascular Cells Is Mediated by Extracellular Vesicles Derived from M1-Activated Microglia

**DOI:** 10.3390/ijms25010015

**Published:** 2023-12-19

**Authors:** Elena Beltramo, Aurora Mazzeo, Massimo Porta

**Affiliations:** Department of Medical Sciences, University of Turin, 10126 Torino, Italy; auroramazzeo89@gmail.com (A.M.); massimo.porta@unito.it (M.P.)

**Keywords:** angiogenesis, diabetic retinopathy, endothelial cells, extracellular vesicles, inflammation, microglia, pericytes, thiamine

## Abstract

The interactions between the neuronal and vascular sides of the retina during diabetic retinopathy (DR) have gained increasing attention. Microglia is responsible for the immune response to inflammation inside the retina, which could be mediated by paracrine signals carried by extracellular vesicles (EVs). We aimed to characterize EVs released from immortalized human microglial cells in inflammation and investigate their effects on the retinal microvasculature and the anti-inflammatory potential of thiamine in this context. M1 pro-inflammatory polarization in microglia was induced through a cytokine cocktail. EVs were isolated from the supernatants, characterized, and used to stimulate human retinal endothelial cells (HRECs) and pericytes (HRPs). Microvascular cell functions and their release of pro-inflammatory/angiogenic factors were assessed. M1-derived EVs showed increased content of miR-21, miR-155, CCL2, MMP2, and MMP9, and enhanced apoptosis, proliferation, migration, and ROS production in HRPs and HRECs. IL-1β, IL-6, MMP9, CCL2, and VEGF release increased in HRPs exposed to M1-derived EVs, while HRECs showed augmented IL-6, Ang2, VEGF, and PDFG-B. Addition of thiamine to M1-microglial cultures reverted most of these effects. In conclusion, M1-derived EVs stimulate functional changes and secretion of pro-inflammatory/angiogenic molecules in microvascular cells, exacerbating inflammatory damage and retinopathy features. Thiamine added to microglia exerts anti-inflammatory effects.

## 1. Introduction

Diabetic retinopathy (DR) affects about one-third of patients with diabetes. Major risk factors for its development and progression are the diabetes duration and the degree of glycometabolic control maintained over the years. Although its annual incidence appears to have decreased in recent years, the rocketing rise of its underlying cause, diabetes, makes this complication an increasing health challenge worldwide [1].

DR has long been considered a microvascular disease; however, in recent years the role of the neuroretina in the earlier stages of this complication has gained major consideration and, nowadays, DR is described as a neurovascular disease [2]. In this context, several different types of cells and their reciprocal interactions are involved. 

As regards the microvascular component of the neurovascular unit, retinal endothelial cells (ECs) and pericytes constitute the main actors [3]. The inner blood–retinal barrier is very similar to the blood–brain barrier, with tight junctions limiting the free passage of solutes and a high ratio of ECs to pericytes (1:1). Thus, pericytes play a major role in controlling EC proliferation and in the interchange and filtration of stimulating/inhibiting factors from the blood to the neuroretina and vice versa. When, in the earlier phases of DR, pericytes are lost, ECs proliferate abnormally, leading to microaneurysms and, ultimately, proliferative DR [3]. Microglial cells are the main cells responsible for the modulation of the immune response to inflammatory stimuli inside the diabetic retina [4]. They respond to inflammation by activating and switching to their M1 pro-inflammatory phenotype, and secreting immune mediators to restore homeostasis [5,6]. Diabetes and DR involve a chronic inflammation status, in which the microglia remains constantly activated (M1 phenotype) and continues to secrete damaging molecules [5], which, in turn, induce the secretion of inflammatory and apoptotic factors by pericytes [7] in a sort of vicious circle. In addition, in DR, the perivascular accumulation of activated microglia is linked to increased vascular permeability [8]. Thus, ECs, pericytes, and microglia are all together involved in a complex interplay.

Extracellular vesicles (EVs) carry several molecules (miRNAs, mRNAs, proteins) and are known to exert a paracrine function in the transmission of signals between neighboring tissues [9,10,11]. Several authors postulated a role for them in the pathogenesis of diabetes and its complications [11,12,13,14,15] and indicated EVs as putative biomarkers of the disease [13,15,16]. Retinal microglia releases EVs that are able to enhance neuroinflammation [17]. We hypothesize that the paracrine signaling between microglia and pericytes/ECs may be mediated by EVs released by microglial cells and carrying pro-inflammatory molecules, which may exacerbate DR.

Thiamine has been described as an antioxidant and anti-inflammatory molecule, with high relevance in counteracting the hyperglycemia-induced metabolic damage in diabetes [18,19]. In murine models, microglial activation was correlated with a deficit in thiamine [20], and its analogue benfotiamine was able to decrease the release of pro-inflammatory factors, while increasing anti-inflammatory mediators in activated microglia [21]. 

In the present study, we characterized EVs obtained by M1 microglia and verified if they induced functional modifications in retinal microvascular cells, comparing these putative effects to those of the direct stimulation of ECs/pericytes with the same M1 cocktail used to induce the activation of microglia. In addition, we assessed the release of inflammatory/angiogenic molecules by pericytes and ECs stimulated with EVs derived from M1 microglia, and the anti-inflammatory potential of thiamine in DR. 

Our hypothesis, subsequently confirmed by the data we obtained with the present work, was that EVs released by M1-activated microglial cells play a pivotal role in the paracrine signaling between microglia and the microvascular cells. These EVs are presumed to carry pro-inflammatory molecules that contribute to the progression of DR. Furthermore, we propose that thiamine, known for its antioxidant and anti-inflammatory properties, has the potential to neutralize the release of inflammatory mediators when added to the M1 cocktail during the stimulation of microglial cells. Our study aims to characterize the EVs obtained from M1 microglia, assess their impact on retinal microvascular cells, and investigate the anti-inflammatory effects of thiamine in the context of DR as a neurovascular disease.

## 2. Results

### 2.1. EV Characterization

According to the Minimal Information for Studies of Extracellular Vesicles (MISEV) guidelines for EV characterization [22], we measured EV size and concentration via NanoSight, and their protein content via Western blotting, choosing markers from the different groups (see Table 3 in [22]). As shown in Table 1, EVs isolated from the supernatants of microglial cells exposed to different treatments showed no difference in size, while a decrease in their supernatant concentrations was observed after the addition of thiamine.

In addition, all EVs expressed the EV markers CD63, CD81 (associated with the EV plasma membrane), and ALIX (a cytosolic EV marker). Apolipoprotein A1 (ApoA1), a lipid contaminant, was not detected, while GM130, which is associated with the secretory pathway, which is not present in EVs, showed a very low expression (see Supplementary Materials, Figure S1). 

### 2.2. EV Expression of Pro-Inflammatory mRNAs and miRNAs

**Figure 1:**
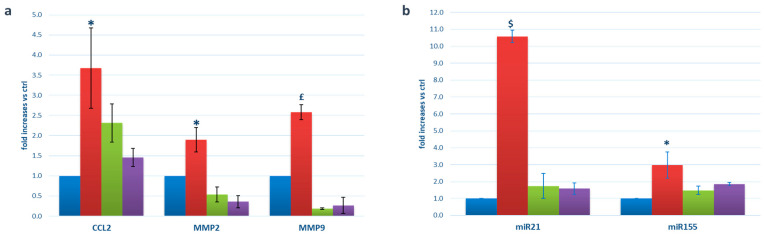
EV expression of pro-inflammatory mRNAs (**a**) and miRNAs (**b**). Blue bars: EVs extracted from the supernatants of microglial cells cultured in physiological conditions (ctrl); red bars: EVs extracted from the supernatants of M1-activated microglial cells (M1); green bars: EVs extracted from the supernatants of microglial cells cultured in the presence of thiamine (T); purple bars: EVs extracted from the supernatants of microglial cells cultured in M1 conditions plus thiamine (M1+T). Mean ± SD of 5 independent experiments. * = *p* < 0.05 vs. ctrl and M1+T; £ = *p* <0.005 vs. ctrl and M1+T; $ = *p* < 0.001 vs. ctrl and M1+T.

We then studied via RT-PCR the expression of specific mRNAs encoding for pro-inflammatory/pro-angiogenic proteins, whose choice was guided by extensive research in the literature (as detailed in Section 3), and our previous research findings [23,24]. We found increased expression of the chemokine (C-C motif) ligand 2 (CCL2), the matrix metalloproteinase-2 (MMP2), and the matrix metalloproteinase-9 (MMP9) in EVs derived from M1-stimulated microglia (Figure 1a), and no variations in vascular cell adhesion molecule-1 (VCAM-1). CCL2 showed a 3.6-fold increase in comparison with EVs derived from unstimulated microglia (ctrl) (*p* < 0.05), MMP2 showed a 1.9-fold increase (*p* < 0.05), and MMP9 showed a 2.6-fold increase (*p* = 0.005). In all cases, the addition of thiamine to the M1 cocktail resulted in a significant reduction in their expression (*p* < 0.05 vs. M1-EVs). In the context of miRNA analysis, we considered our prior results on M1-activated microglia [24] and circulating extracellular vesicles from diabetic patients with diabetic retinopathy [16], in order to identify three miRNAs closely associated with inflammation and angiogenesis (mir21, miR146a, and miR155). We evaluated their expression in EVs, finding 10.5-fold increased concentrations of miR21 (*p* < 0.001 vs. ctrl) and 2.9-fold augmented miR155 (*p* < 0.05) in M1-EVs, and normalization by the addition of thiamine to M1 in microglial cultures (Figure 1b). miR146a did not vary among cases.

### 2.3. Functional Changes in Retinal Microvascular Cells Exposed to Microglia-Derived EVs

**Figure 2:**
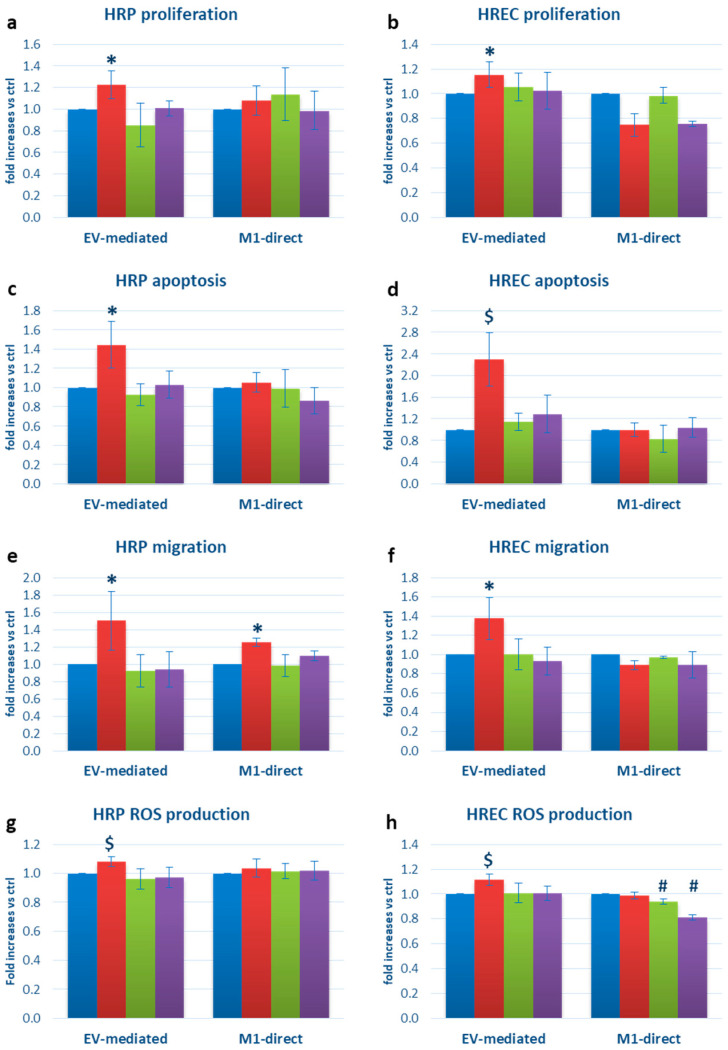
Functional changes in HRPs and HRECs exposed to microglia-derived EVs: (**a**) HRP and (**b**) HREC proliferation; (**c**) HRP and (**d**) HREC apoptosis; (**e**) HRP and (**f**) HREC migration: (**g**) HRP and (**h**) HREC ROS production. Blue bars: cells exposed to EVs extracted from the supernatants of microglial cells cultured in physiological conditions (EV-mediated bars) or directly cultured in physiological conditions (M1-direct bars); red bars: cells exposed to EVs extracted from the supernatants of M1-activated microglial cells (EV-mediated bars) or directly cultured in the presence of the M1 cocktail (M1-direct); green bars: cells exposed to EVs extracted from the supernatants of microglial cells cultured with thiamine (EV-mediated bars) or directly cultured with thiamine (M1-direct bars); purple bars: cells exposed to EVs extracted from the supernatants of microglial cells cultured in M1 plus thiamine (EV-mediates bars) or directly with M1 plus thiamine (M1-direct bars). Mean ± SD of 5 independent experiments. * = *p* < 0.05 vs. ctrl and M1+T; $ = *p* < 0.005 vs. ctrl and M1+T; # = *p* < 0.05 vs. ctrl and M1.

To evaluate the possible effects of M1-EVs on retinal microvascular cell function, we exposed HRPs and HRECs to EVs extracted from microglial cultures in the four different culture conditions. We found around +20% increased proliferation in both cell types (*p* < 0.05 vs. ctrl, i.e., cells exposed to EVs derived from physiological microglia), +44.8% HRP (*p* < 0.05) and +130% HREC (*p* < 0.005) apoptosis, +50.3% HRP and +37.6% HREC migration (*p* < 0.05 in both cell types), and +8.1 HRP and +11.6 HREC reactive oxygen species (ROS) production (both *p* < 0.005 vs. ctrl). The addition of thiamine to the M1 cocktail in stimulating microglia made it possible to normalize all these parameters. By contrast, the direct exposure of HRPs and HRECs to the M1 cocktail resulted in a null influence on apoptosis, proliferation, and ROS production, while slightly increasing HRP migration (Figure 2), thus ruling out the possibility that those changes were due to a direct stimulation of the pro-inflammatory cocktail, rather than a mediation by EVs.

### 2.4. Release of Pro-Inflammatory/Pro-Angiogenic Factors by HRPs/HRECs following Exposure to Microglia-Derived EVs

**Figure 3:**
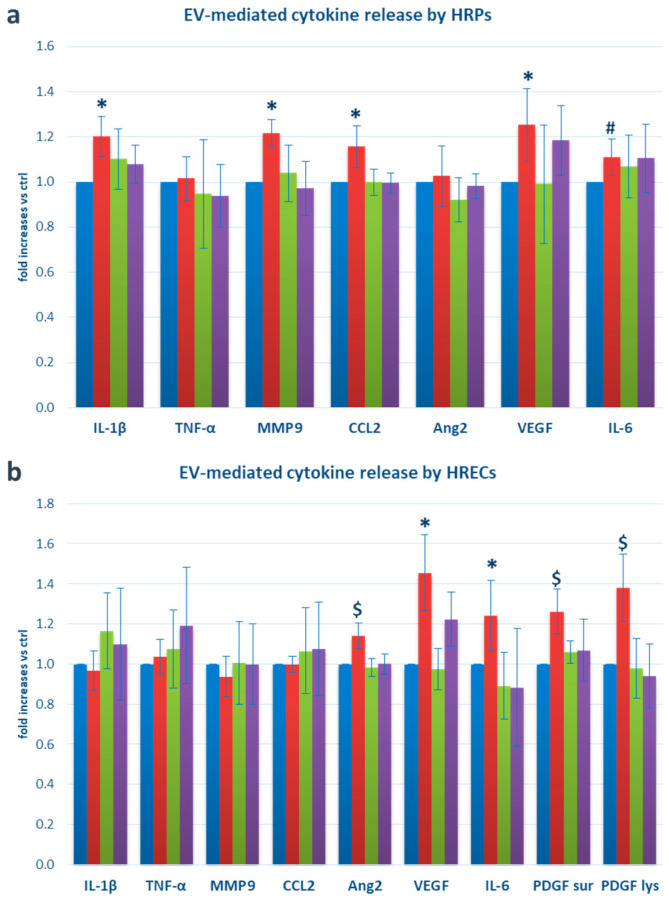
Release of pro-inflammatory/pro-angiogenic factors by (**a**) HRPs and (**b**) HRECs following exposure to microglia-derived EVs. Blue bars: cells exposed to EVs extracted from the supernatants of microglial cells cultured in physiological conditions (ctrl); red bars: cells exposed to EVs extracted from the supernatants of M1-activated microglial cells (M1); green bars: cells exposed to EVs extracted from the supernatants of microglial cells cultured in the presence of thiamine (T); purple bars: cells exposed to EVs extracted from the supernatants of microglial cells cultured in M1 conditions plus thiamine (M1+T). Mean ± SD of 5 independent experiments. * = *p* < 0.05 vs. ctrl and M1+T; $ = *p* < 0.005 vs. ctrl and M1+T; # = *p* < 0.05 vs. ctrl.

Finally, using ELISA, we investigated the release of a panel of pro-inflammatory and/or pro-angiogenic factors by HRPs and HRECs, when exposed to the different types of microglia-derived EVs. We found an increased release of interleukin-1β (IL-1β) (+20.2%, *p* < 0.05 vs. ctrl), interleukin-6 (IL-6) (+10.9%, *p* < 0.05), MMP9 (+21.5%, *p* < 0.05), CCL2 (+15.7%, *p* < 0.05), and vascular endothelial growth factor (VEGF) (+25.4, *p* < 0.05) in HRPs exposed to M1-derived EVs. Tumor necrosis factor α (TNF-α) and angiopoietin-2 (Ang2) were not modified, while platelet-derived growth factor-B (PDGF-B) was undetectable in both supernatants and lysates (Figure 3a). In HRECs, IL-6 showed a + 24.6% release (*p* < 0.05), angiopoietin-2 (Ang2) a +14.1% release (*p* < 0.005), VEGF a +45.5% release (*p* < 0.05), and PDFG-B a +26.1% release in supernatants (*p* = 0.000) and a +38.0% expression increase in lysates (*p* < 0.005) (Figure 3b). The addition of thiamine to M1-microglial cultures reverted most of these effects.

## 3. Discussion

In this study, we demonstrate that the EVs released by activated microglia contain pro-inflammatory and angiogenic mRNAs and miRNAs and induce functional changes in retinal microvascular cells, which, in response, produce and release inflammatory and angiogenic mediators.

Inflammation and abnormal angiogenesis, together with hypoxia, constitute the main players in the cascade of events characteristic of DR. In the earlier phases of the disease, the death and migration of retinal pericytes and the thickening of the basement membrane result in a loss of control of EC proliferation, and in subsequent microvascular abnormalities (microaneurysms) [3]. Later, chronic hypoxia due to an impaired HIF function, together with the increased production of angiogenic molecules, leads to proliferative DR and eventually blindness [25]. In the nervous system and the retina, inflammation is mainly mediated by resident microglia, which can switch between two phenotypes: M2, the resting state, and M1, the activated one, when, in response to damaging stimuli, it acquires mobility and releases pro-inflammatory molecules [26,27]. Once the damaging stimuli are inactivated, the microglia reverts to its M2 phenotype [6]. In the course of DR, the accumulation of harmful metabolites inside the retina causes microglia to remain constantly in its M1-activated state [5], releasing inflammatory cytokines that, in turn, may affect microvascular cells. Pericytes, the first to come in contact with them, in turn release pro-inflammatory and apoptotic mediators [7].

Most studies addressing the microglial potential to modulate DR used rodent cells. However, an immortalized human microglial line, tested for its susceptibility to inflammation, is now available and represents a potential tool to investigate the pathophysiology of the inflammatory component of diabetic retinopathy in species-specific models [24]. We have demonstrated previously that these human microglial cells present major differences from rodent cells in their response to several inflammatory stimuli [24].

EVs are small membrane particles released by most cell types, which may function as carriers of numerous types of molecules (proteins, lipids, mRNAs, miRNAs) through paracrine signaling, thus potentially exerting either a positive or a negative impact on target tissues [9,10,12,15]. In the latest years, they have come into focus as potential biomarkers of several diseases [11,13], including diabetes and DR [14,16], as well as a healing strategy [9,10,11,14,15]. Microglia in the retina releases EVs carrying molecules that are able to exacerbate neuroinflammation [17,28]. Among the many factors stocked inside EVs, miRNAs, small non-coding nucleotide sequences, have particular importance, as they couple with complementary target mRNAs and inhibit their translation into proteins [29]. Freely circulating miRNAs are easily degraded by enzymes, while those carried by EVs are protected by the EV membrane. miRNA concentrations are related to the disease severity, and, like EVs, they are studied as possible biomarkers of cancer and chronic diseases, such as diabetes [30,31] and diabetic retinopathy [16,32].

In our four experimental conditions, EVs released by microglia presented no differences in size, while their concentration was decreased by the addition of thiamine. They all expressed characteristic EV markers, such as CD63, CD81, and ALIX [22]. No contamination with ApoA1 was found, even though collected microglia supernatants contained EV-free bovine serum. A low presence of GM130 accounts for the presence of a small fraction of larger EVs (>200 nm) [22]. 

EVs derived from M1-stimulated microglia showed an increased expression of pro-inflammatory CCL2, MMP2, and MMP9, reflecting in part the content of activated human microglial cells, for which we previously described increments in CCL2, MMP2, and VCAM-1, but not MMP9 [24]. Increased expressions of MMP2 and MMP9 were described in both in vitro models of DR [23] and in the vitreous of patients with proliferative DR [33]. Activated microglia rely on MMPs to facilitate their migration towards the perivascular region, thus increasing vascular leakage [33,34,35], possibly through the detachment of pericytes from the basement membrane, which is in turn enhanced by MMP2 and MMP9 [36]. A high expression of CCL2 is characteristic of neuroinflammation, its release being ascribed to ECs, astrocytes [37], resident microglial cells [38], and pericytes [39]. CCL2 is involved in monocyte and T-cell recruitment, microglia migration, and blood–brain barrier dysfunction [38]. High levels of CCL2 are present in diabetic patients with proliferative DR [40] and macular oedema [41].

In addition, inside M1-derived EVs, we found an increased expression of miR21 and miR155, which are both involved in angiogenesis and inflammation. miR21 is expressed in almost all human cells, particularly in macrophages, monocytes, and dendritic cells [42,43]. In DR, it may modulate inflammation through the downregulation of the peroxisome proliferator-activated receptor alpha (PPARα) and the consequent release of inflammatory factors such as VCAM-1 and CCL2 [44], as well as angiogenesis through the upregulation of MMP9 and HIF-1α, which in turn induces the release of VEGF [44]. We have previously reported increased miR21 in EVs extracted from the plasma of patients with DR and hypothesized a role for it as a potential biomarker of the disease [16,45]. Our present finding may therefore provide further support for a link between the pro-inflammatory and pro-angiogenetic potentials of miR21. The NF-kB-dependent miR155 induction [46] can downregulate the expression of inhibitory proteins of inflammation in microglia activation [47], thus controlling the propagation of inflammation itself [47]. There is evidence that circulating miR155 levels are positively correlated with the severity of DR in diabetic patients [48]. Our findings of increased miR155 expression in M1-EVs are in accordance with those obtained exploring M1 microglia content [24]. By contrast, we did not find an increased expression of miR146a as shown in activated microglial cells [24]. 

Several studies have assessed the role of thiamine as an antioxidant and anti-inflammatory factor, which assumes particular importance in the prevention of metabolic damages caused by hyperglycaemia during diabetes [18,19]. Diabetic subjects often show a relative thiamine deficiency, due in part to renal loss [49]. Reduced thiamine availability is responsible for the increased production of reactive oxygen species (ROS) [19]. Thiamine, and its lipophilic derivative benfotiamine, counteract high glucose-induced damage, reducing ROS production in cell and animal studies, and the progression of DR and nephropathy in diabetic animals and humans [18,50]. Together with its antioxidant properties, a role for thiamine as an anti-inflammatory mediator has been recently hypothesized. A correlation between the progression of microglial activation and thiamine deficiency was observed in a murine model [20], and benfotiamine decreased the expression of inflammatory mediators and increased the production of anti-inflammatory factors in activated murine microglia [21]. We hypothesized that the addition of thiamine to the M1 cocktail when stimulating microglial cells could result in a protection of the vitamin from the release of damaging molecules. Our results show that EVs collected from the supernatants of microglia exposed to the M1 cocktail with the addition of thiamine showed expressions of pro-inflammatory and angiogenic mRNAs/miRNAs superimposable on those of EVs derived from control cultures.

Subsequently, we investigated the effects of these EVs on microvascular cell function and compared them to those obtained with the direct stimulation of pericytes and ECs with the same M1 cocktail. All parameters we considered (proliferation, apoptosis, migration, and ROS production) were increased in both HRPs and HRECs exposed to M1-EVs, while direct stimulation with the M1 cocktail had no effect, apart from increased HRP migration (even though to a lesser extent) also in the presence of M1 stimulation. This stands for a pro-inflammatory and angiogenic paracrine effect mediated by microglia-released EVs, rather than a direct effect of inflammatory mediators. A counterintuitive result was the simultaneous increment of both proliferation and apoptosis. Evidence in the literature, however, demonstrates that apoptotic cells may induce proliferation of neighboring cells through caspases signaling, with important implications for tissue regeneration and wound healing, but also cancer. This is described as “compensatory proliferation” or “apoptosis-induced proliferation” [51,52]. A similar effect was described in vascular smooth muscle cells, due to the differential activation of members of the MAPK family in cell subtypes [53]. The addition of thiamine to the M1 stimulus made the microglial cultures produce EVs that did not affect the microvascular cell functions, demonstrating that, once again, thiamine is a potent inhibitor of damaging effects in these cell types.

Finally, our results show that pericytes and ECs release pro-inflammatory and angiogenic mediators in the supernatants when stimulated by M1-EVs, and that thiamine inhibits this effect when added to M1, confirming the anti-inflammatory potential of this vitamin. Pericytes can react to local neuroinflammation in two different ways: they can release neuroprotective molecules, in an attempt to counteract the damaging insult, or secrete pro-inflammatory factors in a sort of vicious circle, which exacerbates local inflammation [39]. In our experimental setting, HRPs showed increased release in the supernatants of IL-1β, IL-6, CCL2, VEGF, and MMP9, when exposed to M1-EVs. IL-1β is a well-known mediator of inflammation in the retina, secreted mostly by immune cells and generally used to induce a secondary response in pericytes, with the subsequent release of CCL2 and MMPs [39]. Here, we demonstrate that pericytes may respond to the EV-mediated IL-1β+TNFα+IFNγ insult by releasing this cytokine. Our finding of an increased secretion of IL-6, another important mediator of neuroinflammation, by HRPs is confirmed by evidence that pericytes release IL-6 in response to TNFα stimulation to a greater extent than microglia themselves, leading to microglia activation [54]. As already stated, MMP9 increases the permeability of the blood–retinal barrier by degrading the basement membrane and releasing pericytes. Detached pericytes secrete several cito- and chemokines, including CCL2, which facilitates the recruitment of microglial cells in the parenchyma, leading to vascular leakage [39]. At the same time, MMP9 is released by pericytes in inflammatory conditions [39,55], enhancing immune cell infiltration and oedema. The role of VEGF in DR is widely known (reviewed in [56]), and anti-VEGF strategies to block the abnormal retinal capillary leakage and angiogenesis in patients with DR have been extensively studied (reviewed in [57]). An increased VEGF release by HRPs, as a consequence of stimulation with M1-EVs, may therefore worsen DR, together with the enhanced production of Ang2 by HRECs, which induces both pericyte apoptosis [58] and proteolytic degradation of the basement membrane with the release of pericytes [59]. On the other hand, HRECs exposed to M1-EVs show an increased expression and release of PDGF-B, which is usually involved in pericyte recruitment and vascular stabilization [3]. In the very early phases of acute events like ischemia, pericytes may exert a neuroprotective effect via the PDGF-B/PDGFBR axis, by stabilizing new vessels that colonize the necrotic areas [39,60]. Hence, we hypothesize that, in DR, ECs may increment the PDGF release to either counteract the inflammatory insult or stabilize the newly sprouted microvessels. The protective effects of thiamine towards the inflammatory damage, when added to microglia cultures together with the M1 cocktail, is once again confirmed by the findings of no increases in cytokine release by HRPs and HRECs exposed to EVs derived from these cultures.

## 4. Materials and Methods

### 4.1. Cell Cultures

Immortalized human microglial cells and human retinal endothelial cells (HRECs) were purchased from Innoprot (Bizkaia, Spain), and cultured according to the manufacturer’s instructions in microglia medium (Innoprot) or endothelial cell medium (Innoprot), respectively. Human retinal pericytes (HRPs) were immortalized and characterized by our group [61]. They were cultured in DMEM (Thermo Fisher Scientific, Waltman, MA, USA), with 10% added fetal bovine serum.

### 4.2. EV Production and Collection

Human microglial cells were exposed to a cocktail composed by 10 ng/mL TNF-α + 20 ng/mL IL-1β + 50 ng/mL IFN-γ (ThermoFisher), which was demonstrated to be able to induce M1 activation [24]. At the same time, other wells were cultured in physiological conditions (ctrl), 50 µM thiamine (T), or M1+T. The thiamine concentration we used was determined by former dose–response experiments [62,63], and it is acknowledged as the lowest effective dosage in counteracting metabolic abnormalities in diabetic complications [18,50]. After 24 h of exposure, supernatants were discarded and all cultures were kept for a further 24 h in DMEM with 10% fetal bovine serum added, which was fully deprived of EVs by ultracentrifugation. 

Supernatants were centrifuged at 3000× *g* for 30 min to remove debris and apoptotic bodies, followed by ultracentrifugation at 100,000× *g* for 3 h at 4 °C of the cell-free supernatants (ultracentrifuge: Optima L-100K, Beckman Coulter, Brea, CA, USA; rotor: 90 Ti, 90,000 rpm, fixed angle, Beckman Coulter). EVs were either used immediately or stored at −80 °C in DMEM + 5% dimethyl-sulfoxide. The EV size and concentration were measured through a NanoSight LM10 (NanoSight Ltd., Minton Park, UK) running the Nanoparticle Tracking Analysis 2.3 software. For further experiments, we used an EV concentration similar to the one measured in peripheral blood of healthy subjects (8 × 10^8^ EV/mL) [45].

### 4.3. Protein-Content EV Characterization

Protein-content EV characterization was performed in accordance with the MISEV guidelines [17] using Western blotting, choosing markers from the different groups. To extract total proteins, EVs were lysed using M-PER mammalian protein extraction reagent (ThermoFisher) added with a 10 µL/mL protease inhibitor cocktail kit (ThermoFisher). Extracts were kept ice-cold and cleared by centrifugation at 20,000× *g* for 15 min at 4 °C. Protein concentration was determined using the Bradford method. A total of 30 µg of proteins were loaded on pre-cast gels (4–15% Mini-PROTEAN^®^ TGX™ Precast Gel, Biorad, Irvine, CA, USA), separated by electrophoresis, and transferred to nitrocellulose membranes. Specific antibodies to human CD63, CD81, ALIX, ApoA1, and GM130 (Abcam) were used for the immunoblotting of membranes. For specifications of the antibodies and dilutions see Supplementary Materials, Figure S1. The enhanced chemiluminescence (ECL) Western blotting protocol (Merck–Millipore, Burlington, MA, USA) was used to visualize the bands. Each characterization was repeated 3 times.

### 4.4. mRNA Expression

Total RNA was extracted from EVs using the HighPure RNA Isolation kit (Merck). A total of 200 ng of RNA were reverse-transcribed using a High Capacity cDNA Reverse Transcription Kit (Thermo Fisher Scientific). qRT-PCR was performed using a *48*-well StepOne Real Time System (Applied Biosystems, Waltham, MA, USA) using a Power SYBR™ Green PCR Master Mix (Thermo Fisher Scientific). Relative gene expression was determined using the 2^−ΔΔCT^ method and normalized against β-actin. The primers used are listed in the Supplementary Materials, Table S1.

### 4.5. miRNA Expression

Total RNA was extracted from EVs using the mirVana RNA isolation kit (Thermo Fisher Scientific), which also allows for the isolation of small RNAs. RNA was quantified spectrophotometrically (Nanodrop ND-1000, ThermoFisher), and 200 ng of RNA were reverse-transcribed using the miScript Reverse Transcription Kit (Qiagen, Hilden, Germany). qRT-PCR was performed using a 48-well StepOne Real-Time System (Applied Biosystems), using a miScript SYBR Green PCR Kit (Qiagen). Specific primers to miR21, miR146a, and miR155 (Supplementary Materials, Table S2) were used. miRNA expression was normalized against the small nuclear RNA RNU6B.

### 4.6. Cell Function Experiments

HRPs and HRECs were exposed for 24 h to the four types of EVs isolated from microglial cultures, as previously described. Other microvascular cells were cultured in parallel for 24 h in the presence of the M1 cocktail, with or without the addition of thiamine.

Proliferation was assessed using the Cell Proliferation ELISA BrdU kit (Merck), and apoptosis was assessed using the Cell Death Detection ELISA^PLUS^ kit (Merck), according to the manufacturer’s instructions.

Cell migration was measured using the colorimetric QCM Chemotaxis Cell Migration Assay (Merck): cells were seeded inside 8 µm pore polycarbonate membranes and exposed to EVs for 24 h. Subsequently, cells still inside the insert were mechanically removed and those that migrated through the membrane were stained. The dye was then extracted, and colorimetric reading was performed spectrophotometrically at 560 nm.

ROS production was assessed by exposing cells in 96 well/plates for 45 min at 37 °C to 25 µmol/L H_2_DCFDA (Invitrogen–ThermoFisher, Waltham, MA, USA) in medium without red phenol and FCS. Wells were then washed and fresh medium was added. Fluorescence was measured at 490 nm excitation/520 nm emission at different time points.

### 4.7. Release of Pro-Inflammatory/Pro-Angiogenic Factors 

The release of pro-inflammatory or angiogenic molecules in the supernatants/lysates of HRP/HREC cultures exposed to microglia-derived EVs was measured via ELISA, according to instructions. A list of the kits is detailed in the Supplementary Materials, Table S3.

### 4.8. Statistical Analysis

Results are intended as mean ± SD of 5 independent experiments, normalized against a control (EVs obtained from microglia cultured in physiological conditions, or HRPs/HRECs exposed to physiological EVs, or HRPs/HRECs in physiological conditions, as appropriate), unless otherwise stated. Statistical comparisons were carried out using one-way ANOVA with Bonferroni post hoc correction. Results were considered significant for *p* ≤ 0.05. SPSS software version 26.0 (IBM, Armonk, NY, USA) was used for statistical analysis.

## 5. Conclusions

This study addresses the complexity of retinal structures affected by DR, focusing not only on the study of a single cell type or a single district (microvessels vs. neuroretina), but on the interplay between vascular and neuroretinal components. Notably, our model employs exclusively human cells, deviating from the common use of murine cells in microglia studies related to DR. A key aspect of our findings revolves around EVs, which, while serving as informative indicators of cellular health or pathology, present a potential cell-free approach for vessel repair. Our results highlight a concerning feedback loop, where retinal microvascular cells exposed to EVs from M1-activated microglia release in turn pro-inflammatory and angiogenic molecules, intensifying inflammatory damage and contributing to retinopathy progression. The damage induced in microvascular cells by inflammation in our model is mainly due to the paracrine effects of EVs, rather than the direct stimulus of pro-inflammatory molecules.

We also propose thiamine as a promising, non-invasive, and cost-effective therapeutic option. Given the persistent lack of non-invasive therapies for DR—one of diabetes’ most prevalent debilitating complications—thiamine emerges as a potential solution. Its established antioxidant properties offer protection against glucose-induced microvascular damage, and our study further supports its anti-inflammatory potential in the context of DR development. This suggests a compelling avenue for future research, exploring thiamine’s role in preventing or treating this widespread complication.

## Figures and Tables

**Table 1:** Size (nm) and concentration (number × 10^11^/mL) (mean ± SD of 5 independent measures) of EVs derived from human microglia exposed to different stimuli. ctrl = EVs extracted from the supernatants of microglial cells cultured in physiological conditions; M1 = EVs extracted from the supernatants of M1-activated microglial cells; T = EV extracted from the supernatants of microglial cells cultured in the presence of thiamine; M1+T = EVs extracted from the supernatants microglial cells cultured in M1 conditions plus thiamine. * *p* < 0.05 vs. ctrl and M1.

	ctrl	M1	T	M1+T
Size (nm)	181.53 ± 13.23	175.48 ± 15.05	177.83 ± 9.43	183.08 ± 11.67
Concentration (U × 10^11^/mL)	3.04 ± 0.22	3.73 ± 0.41	2.71 ± 0.39 *	2.74 ± 0.51 *

## Data Availability

The original contributions presented in the study are included in the article/supplementary files; further inquiries can be directed to the corresponding author.

## Supplementary Materials

The following supporting information can be downloaded at: https://www.mdpi.com/article/10.3390/ijms25010015/s1.
