# Supplementary material for: Release of Pro-Inflammatory/Angiogenic Factors by Retinal Microvascular Cells Is Mediated by Extracellular Vesicles Derived from M1-Activated Microglia
Source: Int J Mol Sci. 2023 Dec 19;25(1):15. doi: 10.3390/ijms25010015 (PMC10778795; doi:10.3390/ijms25010015)
Supplement: Supplementary file 1 [file ijms-25-00015-s001.zip › ijms-2710043-supplementary.pdf]

Supplementary Material

Figure S1. Protein content-based EV characterization. One/two protein(s) from each category suggested by the MISEV Guidelines 2018 (Théry et al., 2018) were chosen and their presence in EV lysates checked by Western blotting: CD63 (Abcam, ab134045), CD81 (Abcam, ab109201), ALIX (Abcam ab88388), ApoA1 (Abcam, ab52945), and GM130 (Abcam, ab52945). All primary antibodies were rabbit anti-human and diluted 1:1000. Secondary antibody: Goat anti-rabbit IgG (Abcam, ab97051), dilution 1:20000. EV from all experimental conditions were positive for the characteristic EV markers CD63, CD81, and ALIX, negative for the contaminant lipoprotein ApoA1, and slightly positive for GM130.

[1]Théry C. et al (2018). Minimal information for studies of extracellular vesicles 2018 (MISEV2018): a position statement of the International Society for Extracellular Vesicles and update of the MISEV2014 guidelines. *Journal of extracellular vesicles*, *7*(1), 1535750. https://doi.org/10.1080/20013078.2018.1535750

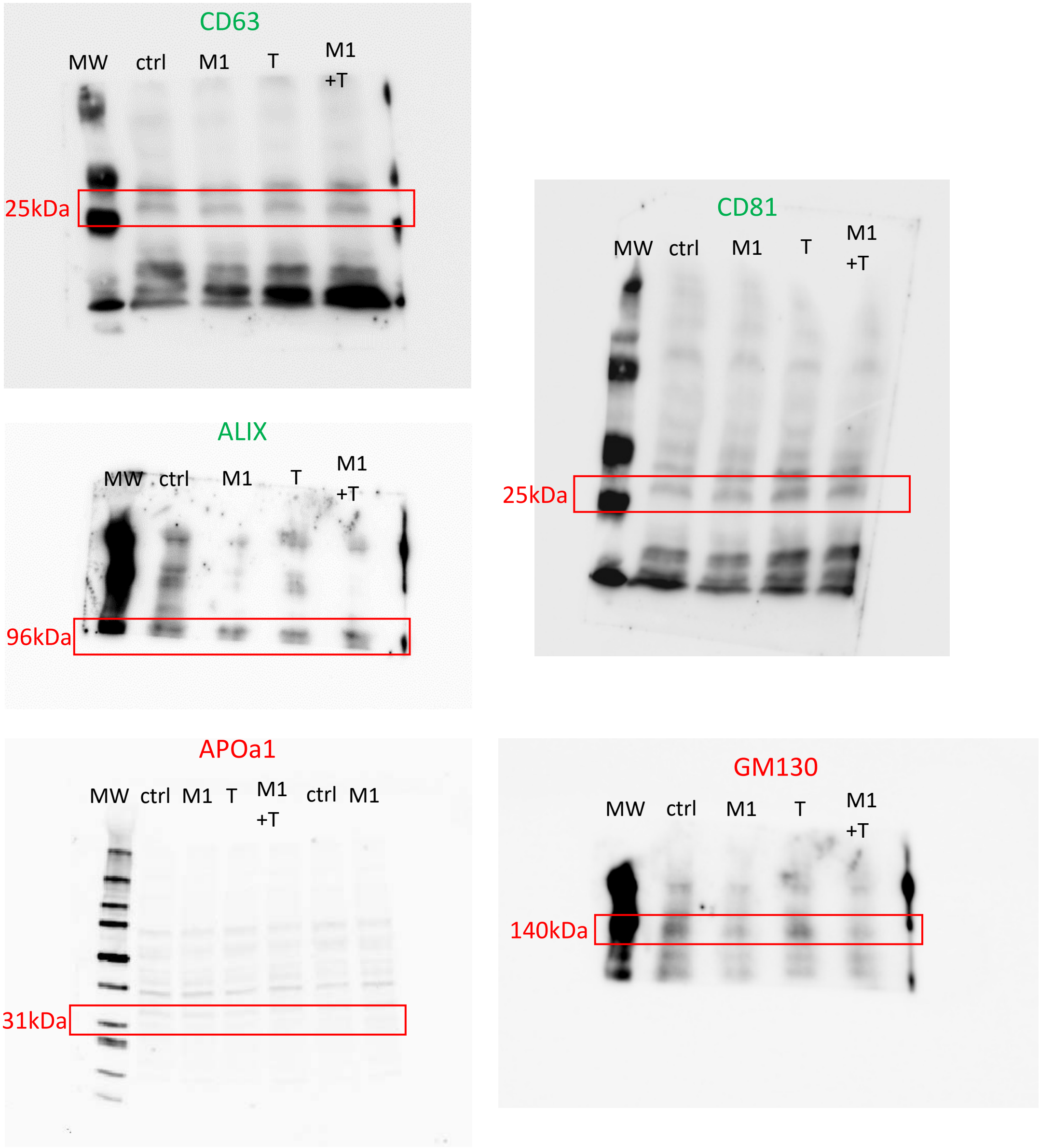

Table S1. Human mRNA primers (Applied Biosystems)

	forward	*reverse*
MMP2	AGCGAGTGGATGCCGCCTTTAA	CATTCCAGGCATCTGCGATGAG
MMP9	CTGAGTCAGCACTTGCCTGTCA	TATGCTTACCCCAGAACCCTCCAAT
VCAM1	CTTTTGGAGTCGAAGATGAGGAAA	CACTACTATCGCAAAACTGACTGAA
CCL2	CCACTTATCACTCATGGAAGATCCC	GAGTAACTGCGCTGAGTGTGTT

Table S2. Human miRNA primers (Applied Biosystems)

miR-21	TAGCTTATCAGACTGATGTTGA
miR-155	TTAATGCTAATCGTGATAGGGGT
miR-146a	TGAGAACTGAATTCCATGGGTT

Table S3. ELISA kits

Kit	*Producer (Distributor)*	*Cat. #*
Cell Proliferation ELISA BrdU	Roche (Merck)	11647229001
Cell Death Detection ELISA^{PLUS}	Roche (Merck)	11774425001
QCM Chemotaxis Cell Migration Assay	Merck	ECM508
IL-1 beta Human ELISA Kit	Invitrogen (ThermoFisher)	BMS224-2
IL-6 Human ELISA Kit	Invitrogen (ThermoFisher)	BMS213-2
MMP9 Human ELISA Kit	Invitrogen (ThermoFisher)	BMS2016-2
MCP-1/CCL2 Human Uncoated ELISA Kit	Invitrogen (ThermoFisher)	88-7399-22
Angiopoietin 2 Human ELISA Kit	Invitrogen (ThermoFisher)	KHC1641
TNF alpha Human ELISA Kit	Invitrogen (ThermoFisher)	KAC1751
VEGF-A Cell Lysates Human ELISA Kit	Invitrogen (ThermoFisher)	EHVEGFACL
VEGF Human ELISA Kit *(supernatants)*	Invitrogen (ThermoFisher)	KHG0111
PDGF-BB (CSRP2) Human ELISA Kit *(lysates)*	Invitrogen (ThermoFisher)	EHCSRP2
PDGF-BB Human ELISA Kit *(supernatants)*	Invitrogen (ThermoFisher)	BMS2071
